# Candida-Related Immune Response Inflammatory Syndrome Treated with Adjuvant Corticosteroids and Review of the Pediatric Literature

**DOI:** 10.4274/tjh.2016.0237

**Published:** 2017-03-01

**Authors:** Dildar Bahar Genç, Sema Vural, Nafiye Urgancı, Tuğçe Kurtaraner, Nazan Dalgıç

**Affiliations:** 1 Şişli Hamidiye Etfal Training and Research Hospital, Clinic of Pediatric Oncology, İstanbul, Turkey; 2 Şişli Hamidiye Etfal Training and Research Hospital, Clinic of Pediatric Gastroenterology, İstanbul, Turkey; 3 Şişli Hamidiye Etfal Training and Research Hospital, Clinic of Pediatrics, İstanbul, Turkey; 4 Şişli Hamidiye Etfal Training and Research Hospital, Clinic of Pediatric Infectious Disease, İstanbul, Turkey

**Keywords:** Leukemia, Febrile neutropenia, Candida, Immune response inflammatory syndrome

## TO THE EDITOR,

Chronic disseminated candidiasis (CDC) is a potentially fatal complication observed in febrile neutropenia [1]. The diagnosis is usually made after neutrophil recovery and microbiological proof has been often negative [2]. Granulomatous histopathology, radiological lesions coincident with resolution of granulocytopenia, and rapid response to corticosteroids favors immune-mediated pathogenesis. Recently, CDC has been suggested to be related to Immune response inflammatory syndrome (IRIS), an exacerbated response to a preexisting antigenic stimulus in patients with rapid immune restoration [1,3]. IRIS has been mostly documented in HIV-infected patients with immune recovery after antiretroviral therapy [4]. Here, we present a case of *Candida*-related IRIS and review the current literature on children.

**Figure 1:**
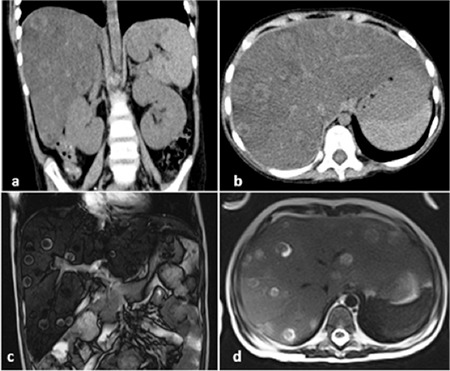
Coronal and axial computed tomography images (a, b); coronal and axial magnetic resonance images of circumscribed typical hepatic Candida lesions (c, d).

A male, aged 6 years and 7 months, with B-cell acute lymphoblastic leukemia was treated for presumed typhlitis with meropenem, teicoplanin, and amphotericin B during induction therapy. Thoracoabdominal CT scans revealed hepatosteatosis/hepatomegaly. Fever subsided on the 2^nd^ day. During steroid tapering and on the 8^th^ day of antibiotics, the patient developed fever and abdominal pain with marked elevation of liver enzymes, predominantly of GGT. Bone marrow examination showed no evidence of blasts or hemophagocytosis and the blood count was normal. Control imaging showed typical widespread hepatic bull’s eye lesions (Figure 1). The liver biopsy demonstrated granulomatous inflammation, but no fungus was detectable. According to European Organization for Research and Treatment of Cancer/Mycoses Study Group criteria, the diagnosis was possible invasive fungal infection, most likely candidiasis. Reappearance of symptoms after neutrophil recovery indicated IRIS. We empirically administered dexamethasone for 14 days. Fever disappeared after 24 h and liver function tests improved in 1 week. He was discharged with oral voriconazole. During vincristine therapy, voriconazole was replaced with amphotericin B to avoid toxicity. In the 13^th^ month of voriconazole, the liver lesions showed partial regression and calcification. As re-biopsy was negative for microorganisms and showed only rare microgranulomas, we stopped the voriconazole. The patient completed chemotherapy and has been without any exacerbation for 32 months since the initial diagnosis of IRIS.

Clinical and/or radiological deterioration after neutrophil recovery is a well-known entity in patients treated for opportunistic infections [4]. The immune system shifts towards Th-1 type response and amplifies proinflammatory cascades [1]. Therefore, the severity of radiological/clinical findings might depend on the immune status of the patient [5,6]. IRIS is a diagnosis of exclusion; other possible causes of persistent fever should be evaluated. If the clinical scenario is not consistent with preexisting disease, treatment side effects, or a possible newly acquired pathogen, IRIS deserves diagnostic consideration. In the previous *Candida*-related IRIS reports on children with cancer, all patients had fever and liver dysfunction accompanying normal neutrophil counts. Liver biopsies showed granuloma formation. Tissue cultures for fungi were negative in all samples except one. The most commonly administered antifungal agent was amphotericin B. Details of steroid therapy and the outcomes are presented in Table 1 [3,7,8,9,10,11]. Increased susceptibility to infection might be a drawback for prolonged corticotherapy. However, neither *Candida* reactivation nor other new opportunistic infections have been reported [3].

*Candida*-related IRIS has been rarely reported in children. Early recognition and appropriate management of IRIS might prevent unnecessary diagnostic procedures, antibiotic usage, and chemotherapy delays.

## Figures and Tables

**Table 1:**
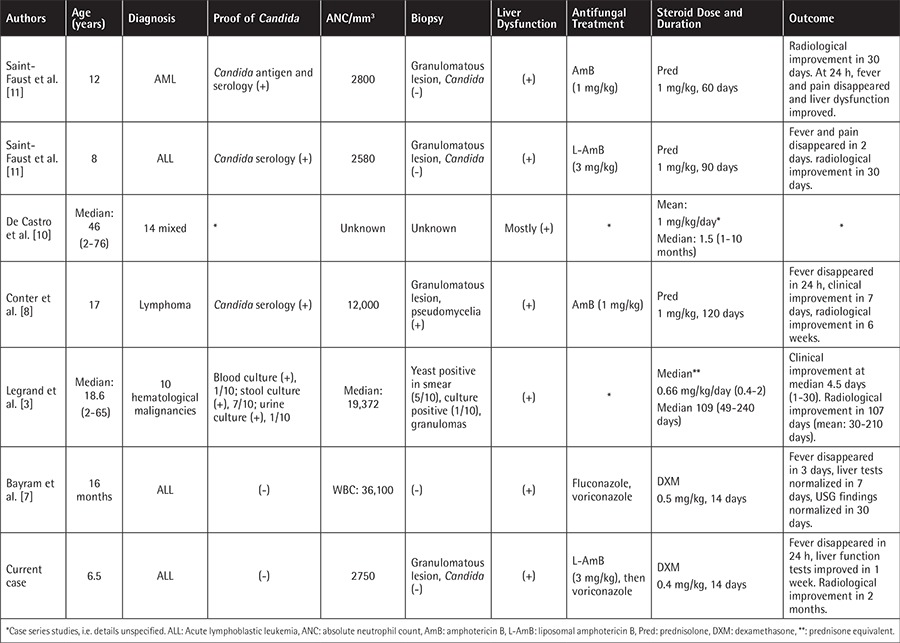
Review of pediatric cases of Candida-related Immune response inflammatory syndrome treated with corticosteroids.

## References

[1] Rammaert B, Desjardins A, Lortholary O (2012). New insights into hepatosplenic candidosis, a manifestation of chronic disseminated candidosis. Mycoses.

[2] Fleischhacker M, Schulz S, Jöhrens K, von Lilienfeld-Toal M, Held T, Fietze E, Schewe C, Petersen I, Ruhnke M (2012). Diagnosis of chronic disseminated candidosis from liver biopsies by a novel PCR in patients with haematological malignancies. Clin Microbiol Infect.

[3] Legrand F, Lecuit M, Dupont B, Bellaton E, Huerre M, Rohrlich PS, Lortholary O (2008). Adjuvant corticosteroid therapy for chronic disseminated candidiasis. Clin Infect Dis.

[4] Manabe YC, Campbell JD, Sydnor E, Moore RD (2007). Immune reconstitution inflammatory syndrome: risk factors and treatment implications. J Acquir Immune Defic Syndr.

[5] Karthaus M, Huebner G, Geissler RG, Heil G, Ganser A (1998). Hepatic lesions of chronic disseminated systemic candidiasis in leukemia patients may become visible during neutropenia: value of serial ultrasound examinations. Blood.

[6] Pestalozzi BC, Krestin GP, Schanz U, Jacky E, Gmür J (1997). Hepatic lesions of chronic disseminated candidiasis may become invisible during neutropenia. Blood.

[7] Bayram C, Fettah A, Yarali N, Kara A, Azik FM, Tavil B, Tunc B (2012). Adjuvant corticosteroid therapy in hepatosplenic candidiasis-related iris. Mediterr J Hematol Infect Dis.

[8] Conter CD, Thiesse P, Bienvenu A (2007). Persistent fever and hepatosplenic candidiasis, efficiency of a corticoid therapy. J Mycol Med.

[9] Chaussade H, Bastides F, Lissandre S, Blouin P, Bailly E, Chandenier J, Gyan E, Bernard L (2012). Usefulness of corticosteroid therapy during chronic disseminated candidiasis: case reports and literature review. J Antimicrob Chemother.

[10] De Castro N, Mazoyer E, Porcher R, Raffoux E, Suarez F, Ribaud P, Lortholary O, Molina JM (2012). Hepatosplenic candidiasis in the era of new antifungal drugs: a study in Paris 2000-2007. Clin Microbiol Infect.

[11] Saint-Faust M, Boyer C, Gari-Toussaint M, Deville A, Poiree M, Weintraub M, Sirvent N (2009). Adjuvant corticosteroid therapy in 2 children with hepatosplenic candidiasis-related IRIS. J Pediatr Hematol Oncol.

